# RETRACTION: Dermoscopic and Reflectance Confocal Microscopic Features of Children Scabies

**DOI:** 10.1111/srt.70211

**Published:** 2025-07-22

**RETRACTION**: Z. Guan, T. Bi, and Q. Li, “Dermoscopic and Reflectance Confocal Microscopic Features of Children Scabies,” *Skin Research and Technology* 29, no. 9 (2023): e13459, https://doi.org/10.1111/srt.13459.

The above article, published online on 7 September 2023 in Wiley Online Library (wileyonlinelibrary.com), has been retracted by agreement between *Skin Research and Technology*; and John Wiley & Sons Ltd. The article was accepted for publication following an insufficient peer review process that does not meet the standards of the journal's peer review policy. Furthermore, scientific inconsistencies between methodology described and results presented were found. As a result, the editorial team cannot vouch for the reliability of the study's conclusions. Accordingly, the article has been retracted. The authors have been informed of this decision and disagree with it.

